# Unpacking vertical and horizontal integration: childhood overweight/obesity programs and planning, a Canadian perspective

**DOI:** 10.1186/1748-5908-5-36

**Published:** 2010-05-17

**Authors:** Lynne M MacLean, Kathryn Clinton, Nancy Edwards, Michael Garrard, Lisa Ashley, Patti Hansen-Ketchum, Audrey Walsh

**Affiliations:** 1 Community Health Research Unit, University of Ottawa, 451 Smyth Road, Ottawa, Ontario K1H 8M5, Canada; 2 Nursing, Epidemiology and Community Medicine, University of Ottawa, 451 Smyth Road, Ottawa Ontario K1H 8M5, Canada; 3 Maternal Child Health Program, First Nations and Inuit Health Branch, Health Canada, Ottawa K1A 0G5, Canada; 4 Nursing Best Practices Research Unit, University of Ottawa, 451 Smyth Road, Ottawa, Ontario K1H 8M5, Canada; 5 Canadian Nurses Association, 50 Driveway, Ottawa, Ontario K2P 1E2, Canada; 6 School of Nursing, St. Francis Xavier University, PO Box 5000 Antigonish, NS B2G 2W5, Canada; 7 Faculty of Nursing, University of Alberta, 3rd Floor Clinical Sciences Building Edmonton, Alberta, T6G 2G3, Canada; 8 Department of Nursing, Cape Breton University, PO Box 5300, Sydney, NS B1P 6L2, Canada

## Abstract

**Background:**

Increasingly, multiple intervention programming is being understood and implemented as a key approach to developing public health initiatives and strategies. Using socio-ecological and population health perspectives, multiple intervention programming approaches are aimed at providing coordinated and strategic comprehensive programs operating over system levels and across sectors, allowing practitioners and decision makers to take advantage of synergistic effects. These approaches also require vertical and horizontal (v/h) integration of policy and practice in order to be maximally effective.

**Discussion:**

This paper examines v/h integration of interventions for childhood overweight/obesity prevention and reduction from a Canadian perspective. It describes the implications of v/h integration for childhood overweight and obesity prevention, with examples of interventions where v/h integration has been implemented. An application of a conceptual framework for structuring v/h integration of an overweight/obesity prevention initiative is presented. The paper concludes with a discussion of the implications of vertical/horizontal integration for policy, research, and practice related to childhood overweight and obesity prevention multiple intervention programs.

**Summary:**

Both v/h integration across sectors and over system levels are needed to fully support multiple intervention programs of the complexity and scope required by obesity issues. V/h integration requires attention to system structures and processes. A conceptual framework is needed to support policy alignment, multi-level evaluation, and ongoing coordination of people at the front lines of practice. Using such tools to achieve integration may enhance sustainability, increase effectiveness of prevention and reduction efforts, decrease stigmatization, and lead to new ways to relate the environment to people and people to the environment for better health for children.

## Background

### The importance of vertical and horizontal integration in childhood overweight/obesity interventions

Increasingly, multiple intervention programming has been suggested as a key approach to developing public health initiatives and strategies [1,2]. Using socio-ecological and population health perspectives, multiple intervention program approaches endeavour to provide coordinated and strategic comprehensive programs operating over system levels and across sectors, allowing practitioners and decision makers to take advantage of synergistic effects. These approaches also require vertical and horizontal (v/h) integration of policy and practice in order to be maximally effective. This paper examines v/h integration of interventions for childhood overweight/obesity prevention and reduction, given the complex and multi-level nature of obesity, including environmental, social, community, organizational, and policy system levels.

In the past, obesity prevention and treatment programs have typically focused on health education and individual behaviour change, with emphasis on personal lifestyle and responsibility. Yet, advances in socio-ecological thinking over the last decade point to system change as the missing link in addressing the obesity increase [3,4].

Systemic environmental influences relate to socio-ecological features of the problem and include individual, home, school, community, national, and international components [5]. As noted in the Doak *et al*. [5] review of child and adolescent obesity prevention programs, a wide array of multi-level factors have impact on the prevalence of overweight/obesity. For example, at the school, community, and national levels, environmental influences can include the school curriculum, transportation system, socio-economic status of aggregate populations, community recreation opportunities, community attitudes, imported and local goods, the economy, and the price and availability of food [6]. There has been a call for a less medical, more preventative, public health approach to childhood obesity that focuses on upstream, more distal causes and interventions for prevention [7-9]. Such a complex problem crossing many system levels would benefit from an integrated approach to intervention.

### Key concepts

Key concepts for this paper include intervention, synergy, sector, intersectoral collaboration, and v/h integration. To clarify, we are not referring to the discussion of whether specific health problems should be dealt with separately or integrated with other health problems in a service delivery model [10,11]. Rather, we are looking at the integration of a system of players, policies, and programs within jurisdictions and across one or more related health issues, in order to maximize people's wellbeing.

For our discussion here, an intervention is a single public health activity meant to positively affect the health of target groups [12], whether that be aimed towards prevention, control, or reduction of negative conditions, or enhancement or maintenance of positive ones. Multiple intervention programs are organized, funded sets of interventions with coordinated, interconnected intervention strategies targeting at least two different levels of a system (*e.g*., individual behaviour change; organizational change; municipal by-law change) even if each level has only one intervention [12]. Such programs are based on socio-ecological models that attest that health is determined by complex interactions between behavioural, biological, cultural, social, environmental, economic, and political factors. Determinants do not work independently but interact, and may mitigate or compound the effects of other determinants. Effective population health approaches often reflect a socio-ecological framework [1,12,2].

Synergy is the interaction of two or more interventions, such that their combined effect is greater than the sum of their individual effects [12].

The term 'sector' is often used to describe the division of organizations along economic lines into three major sectors: public, private, and non-profit [13]. Other common uses of the term include describing different government ministries within the same level (*e.g*., federal or provincial Ministry of Health, Ministry of Education) as well as describing communities of interest based on issue content (education, housing, public health). However, in this article we will be using 'sectors' to mean issue-based entities (*e.g*., education), because this can include private, and non-profit organizations, as well as public ones with specific jurisdictions.

V/h integration refers to combining and coordinating efforts over multiple system levels, as well as across sector levels within the same system level [14,15]. Integration has structural components (such as a framework of aligned groups, policies, and goals) and process components.

Inter-sectoral collaboration is a term often used for integrated initiatives where both horizontal and vertical dimensions are key [16]. We are using vertical integration in the Canadian sense, where for example multiple levels of government (municipal, regional, provincial, and federal) need to coordinate their efforts. When rapid responses and time-limited approaches are required, vertical integration of programs are effective [17].

We are using horizontal integration to describe the engagement of several sectors (*e.g*., health, education, agriculture, justice) at the same level. In Canada, horizontal integration occurs, for example, when one federal ministry becomes the lead agency of several federal ministries who work together to provide programs, policies, and research in an area of common interest and overlapping accountability. The purpose of horizontal integration is to increase capacity, maximize resources, and minimize duplication of effort [15].

Combining vertical or horizontal approaches may have benefits when the health issue is complex, requiring a multi-sectoral response that spans both governmental and non-governmental actors [18]. Benefits from adding vertical integration include: enhanced opportunities for sustainability; opportunity to work with more of the underlying determinants; prevention of negative spinoff effects for health systems and non-targeted populations; decreased duplication of services; and pooling of funding or resources [17]. If horizontal integration is involved, key factors that are operating simultaneously in the various contexts of children's lives are more likely to be included. For example, if only the health sector is involved, important issues in education, community involvement, and social welfare may be ignored. Horizontal involvement brings opportunities to develop complementary, supportive, synergistic programs and policies. Furthermore, other programs and policies in these sectors that may work in opposition to the health initiative are more likely to be identified or modified.

With solely horizontal approaches, intervention discussions may remain at either the policy level across sectors or at the service delivery sector, without attention to differing levels of jurisdiction [17]. When vertical integration is not part of the picture, important opportunities to provide consistent inter-sectoral policy regulation and resources may be lost. Different kinds of interventions, with targets ranging from broad social determinants of health dealt with at a federal level, down through provincial and municipal levels may not be provided in a consistent fashion. For example, the provision of tax deductions for fees paid by parents for children's sports and fitness activities in Canada is a federal government initiative meant to increase accessibility to active living. Its effects would be undermined if, at the municipal level, cities raised user fees for sports and recreational activities and venues.

Besides the additive advantages of combining v/h integration, there is also the possible advantage of producing synergistic results. Such results could occur across system levels and sectors, in terms of the impacts of the various staged, strategic interventions, the development of committed initiative teams, and the potential spread of salience of the issues and interventions beyond those immediately involved [1]. Most multiple intervention programs rely on the effect of synergy that should come as result from the combined presence of both types of integration [1,17].

The findings on complex programs involving v/h integration have been mixed, partly due to a variety of methodological difficulties in evaluating multiple components, providing interventions of sufficient breadth and strength, lack of sufficient penetration and reach in communities, lack of theoretical underpinnings, and by insufficient intervention in policy and regulation [2]. However, the HIV/AIDS work in Africa is often cited as an example of v/h integration of complex and multiple interventions showing success [2,18]. In Kenya, for example, interventions have occurred in the areas of health policy, education for individuals, schools, and communities; increased accessibility to treatment and management, infrastructure to support same, and counselling and social support for families [19]. Part of a national framework, these activities were aimed at national, provincial, district, community, household, and individual levels, and involved people from the public, private, civil, and community sectors [19]. Improvements in HIV/AIDS transmission rates and treatment accessibility have been attributed to this coordinated response. The Sub-Saharan African countries, including Kenya, have National AIDS Commissions-coordinating bodies, often sitting outside the Ministry of Health that work with creating and maintaining v/h integration, among other things. In an evaluation of all the National AIDS Commissions in this region, Morah and Ihalainen conclude that, by and large, these commissions have worked well in providing multi-sectoral coordination, strong leadership, advocacy for national frameworks, and engagement of non-governmental actors [18].

However, the commissions have had their difficulties. Issues relevant to this paper, beyond the commissions' unique structures and relationships to government, suggest that the process of maintaining v/h integration is important. Challenges in process include monitoring and evaluation of interventions, and difficulty reaching and acting on decisions quickly, due to an accompanying lack of authority and accountability.

Thus, various functions and processes are required to keep integrated programs and policies cohesive, coordinated, and evolving towards their goals and objectives. Positive and beneficial alignments require more than common goals; to be maintained and function properly, they require information flow and communication among and over levels, as well as coordination, compromise, and sharing boundaries. Without coherence in decisions over levels, not only may integration not work, but the system may lose authority and legitimacy [20].

For example, programming at the community or individual level should be supported by provincial and national activities. Progress toward a goal is enhanced by a common understanding of the problem and of the strategies to address it. Strategies are complementary to and support each other and build on each other. Communication among different levels is such that each jurisdiction can see how its role fits into a coordinated continuum of services, with mechanisms in place to identify and address any deviation from goals and functions. This communication and its feedback mechanisms help to plan integration, establish workable processes, and identify when the integration is not working.

When developing policy and practice involving v/h integration, several considerations are central. Including both horizontal and vertical levels are important for program success, as this maximizes reinforcing and synergistic effects [12]. Including all the key players is also critical. Communication and feedback about system components, their coordination, and effectiveness are important [14], and structures are necessary for planning, designing, and monitoring [21]. Coherence in decisions, plans, goals, and processes is the underlying purpose of this complex undertaking, and must be maintained [22]. Finally, relationship building and maintenance are key to integration effectiveness over time and players [23].

The question then becomes, what does v/h integration look like? What does it look like in the area of childhood overweight/obesity intervention? And what are the implications for research, practice, and policy?

## Discussion

### Childhood overweight/obesity and vertical/horizontal integration

As childhood overweight/obesity becomes more pervasive, and the indications of its complex, multi-level sources of causation become more apparent [5,3,4], the need for a multiple intervention program approach for policy and practice in the area is more evident. And this call for multi-level, multi-sector intervention requires the involvement of many different sectors in an integrated and targeted fashion.

The sustainability of intervention impact for obesity is a critical problem faced by practitioners. Short-term behavioural interventions seem not to be effective, particularly in the absence of complementary interventions that address sustainability and foster an environment that is supportive of long-term behavioural modification and societal level change [15,24]. In particular, a key recommendation of studies of school-based programs is that a broader involvement of stakeholders (educators, community, parents, and students) is needed to bring about a sustainable impact. An important implication of this recommendation is that effective obesity interventions in the school setting require corresponding and linked interventions at the family and community levels. This lends support to our premise that both vertical and horizontal integration are critically required aspects of effective childhood obesity prevention programs. Challenges to instituting v/h integrated approaches in Canada may be health funding structures, which may make it difficult to work over provincial and federal levels. The federal government, setting broad health policy, gives funds to the provincial governments who are responsible for direct provision of services. Another factor may be discouragement for government and publicly-funded groups to work outside their mandates. McLaren *et al*. [15] recommend a reward system for cross-sectoral engagement, or appointment of a specific public health committee across government sectors [15].

Some obesity investigators report 'there is no consistent, compelling portrait in favour of vertical integration' [15]. These same investigators suggest, however, that this may be due to the limitations of randomized controlled trial (RCT) and control group studies they examined, the behavioural theoretical nature of many integration approaches suggested, and the virtual absence of 'upstream' level factors incorporated into intervention approaches. Another limitation to this conclusion is that the interventions they examined tended not to go vertically beyond community level. Indeed, McLaren *et al*. [15] do call for intervention at the policy level and at the larger social determinants level. The systematic examination of horizontal integration has had little study to date, but has been called for [15]. Although we echo their recommendations for inclusion of further levels into vertical integration, we suggest that the evidence from socio-ecological systems approaches using literature beyond RCT reviews points to the usefulness of vertical integration, although not in isolation of horizontal integration [25,5-28]. We will review examples of successful v/h integration in the understanding of childhood overweight/obesity intervention and explore some of the implications of these approaches for policy, research, and practice.

When the childhood obesity literature was examined, few studies were found that mentioned consideration of, or action related to, integration, in the way we use it. Several, however, identified the importance of factors related to integration and urged that future intervention research and implementation include multiple levels of influence [8]. This becomes significant given that there is currently no firm evidence to support any specific intervention approach to childhood overweight/obesity prevention, particularly of the single intervention type [25]. There is more evidence supporting the use of multi-faceted approaches that address both physical activity and nutritional issues [5,26,27].

Literature suggests consistent links and synergies between and among individual, family, and community-based interventions may enhance the success of prevention initiatives [29,11]. Increasing involvement of decision makers and policy makers would also be useful by enhancing links and synergies among sectors. Further, enhanced horizontal and vertical integration may, in turn, enhance sustainability [12] through stakeholder buy-in. However, as helpful as socio-ecological approaches are in conceptualizing the issue, their emphasis is typically across system levels. Evidence suggests that coordinated interventions across sectors and within levels may also be important elements to prevention, control, and reduction of overweight/obesity in children. For example, in their review of the literature, McLaren *et al*. [15] call for incentives supporting intersectoral integration in government, regulation of advertising and promotion of food to children, and fiscal policies to support healthy lifestyles, among others.

As interventions move to address the different settings of the overweight/obese child's life (home, school, health system) and over different system levels (school, community, physical and economic environments), it becomes important to look at how those layers can support each other. Considerations of v/h integration of policy and practice become significant.

### Vertically and horizontally integrated childhood overweight/obesity initiatives

Examples of successful intersectoral, integrated approaches to childhood overweight/obesity, though few, are emerging with promising results. For the most part, these initiatives are relatively recent, and their final effectiveness is as yet unknown, but they do point out some of the issues that arise when integration is attempted across both horizontal and vertical dimensions. Notable examples are Action Schools! BC [30,31], Calgary Health Region's Community Prevention of Childhood Obesity Program [32], the Strategic Alliance for Healthy Food and Activity Environments [33], the Consortium to Lower Obesity in Chicago Children [34], and Arkansas' comprehensive initiative to combat childhood and adolescent obesity, based on a cross-sector approach that involves vertical integration of legislative powers at the state level [35].

A Canadian initiative, Action Schools! BC, is briefly described next to provide an overview of how v/h integration works and its importance to childhood obesity prevention programs. British Columbia's healthy schools initiative, Actions Schools! BC, is based on a socio-ecological model and has implemented a school-based physical activity and healthy eating program that was initially aimed at elementary school children, and later expanded to include high school students. The program is focused on creating school environments where students are given many new opportunities to make healthy choices. Supportive community and provincial environments have provided the resources and political investment required to ensure program uptake and sustainability. An assessment of a 17-month pilot of this multilevel, partnership-based approach at the provincial and local levels found policy development and funding and regulation changes that were attributed, although not definitively, to the Action Schools! BC model and its implementation. The researchers concluded that the environment for school and provincial action on health behaviours improved, and that influential factors included political will and public interest [31].

Partnerships were formed horizontally across sectors (health, education, tourism, sports, and relevant disciplines) and vertically, from practitioners to decision makers within the education sector. This integration was accomplished through three committees: a provincial advisory committee (core community, school, and government stakeholder representatives) that was horizontally integrated across evaluation and support teams and vertically integrated among education stakeholders; the AS! BC support team that assisted school advisory committees; and a multidisciplinary evaluation team [31]. The pilot evaluation included both outcome and process measures. The outcome evaluation found that students in the intervention schools had increased physical activity levels, heart health, bone health, dietary requirement assessment, and academic performance. No differences were found for body mass index (BMI), fruit and vegetable consumption, and psychosocial variables such as self esteem and motivation. The process evaluation found that administrators, teachers, and parents were very satisfied with the program model and supported wider implementation [30]. The combined success of program integration, both horizontally across sectors and vertically over the levels of the education system (from school to province), and the pilot results convinced the provincial government to introduce the program province-wide [31]. This is a unique initiative that points to the link between v/h integration and program effectiveness.

### A conceptual framework

The more complex a set of interventions, the more likely it is that a conceptual framework is necessary to help define policy issues, practice requirements, and determine processes and outcomes for research and evaluation. Indeed, this is the rationale behind logic models and other planning tools. Multiple intervention programs conducted over system levels and sectors require such frameworks to guide planning, intervention, and evaluation [12].

**Figure 1:**
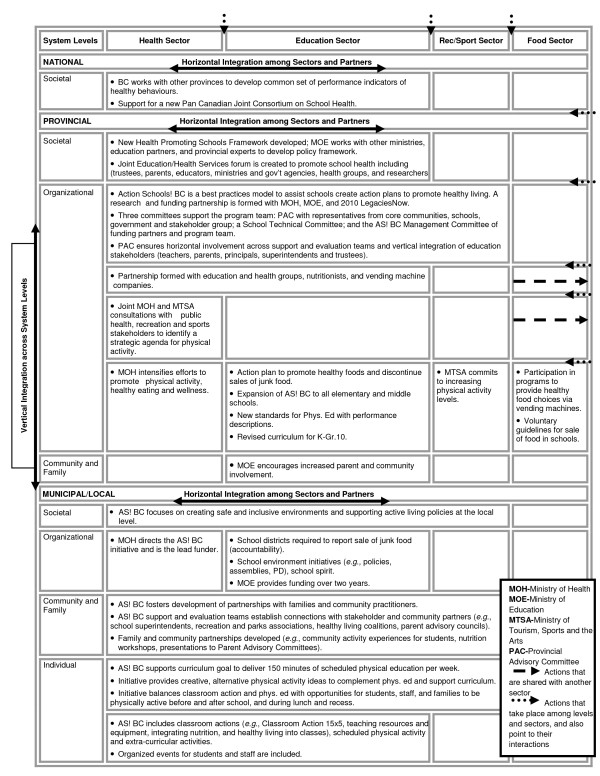
**An Integrated Intersectoral Intervention Framework**: BC's Comprehensive Plan to Support Student Health, with a focus on Action Schools! BC Childhood Obesity Prevention Initiative (Source: *Intersectoral Action Towards Population Health*, Public Health Agency of Canada, June, 1999 Adapted and Reproduced with the permission of the Minister of Public Works and Government Services Canada, 2008.)

We have adapted a two dimensional framework for multi-level program integration and applied it to the Action Schools! BC program (as an example of a v/h integrated childhood obesity program) to illustrate how v/h integration functions and to illuminate issues for discussion. The original framework derives from the report, Intersectoral Action ... Toward Population Health [36]. The two dimensions are: a horizontal dimension linking different sectors or broad levels of activity or categories of partners across one level; and a vertical dimension, which links different levels defined by geography, government levels, or organizational levels within individual sectors. Creators of the original framework felt that including both dimensions, as well as all key players, is critical to the success of such initiatives, as it maximizes 'reinforcing and synergistic effects' [36]. As can be seen in Figure 1, we have further adapted this framework to include the system levels (societal, organizational, community, family, individual) at which intervention policy and practice can be aimed.

The arrows at the top and right side of the chart point to the 'space' between sectors and levels. They are there to draw attention to the interaction and linkages among sectors and levels. These interactions and linkages indicate the processes of the actions. One of the critical challenges of managing programs that are based on collaboration with multiple sectors, partners, and government/organizational levels is to work effectively at the boundaries, so-called 'boundary management.' It is our view that the framework, though a very helpful presentation of structure, still requires some modification. It neither captures the dynamic nature of the interaction among levels, nor how a change in one level or cell may impact or shift others. Shifts can occur as a result in a change in the context (an unexpected global epidemic requires refocusing of health dollars away from obesity intervention), the boundaries (*e.g*., funding changes result in a loss of key coordinating people for service delivery-focused agencies;), across sectors (new policy agencies are created), or even as a result of the intervention itself (success in intervening at one level of the system leads to drawing back of funding for another). A future direction is to develop new ways to capture these extra layers of complexity to make a useful framework even more effective.

### Implications for research, policy, and practice

Obesity in children calls out for application and evaluation of intervention approaches addressing multiple causes at multiple system levels. At the very least, the issue requires policy and programming that do not undermine what other jurisdictional levels and sectors are attempting to do in their own spheres. Optimally, it requires working in a strategic and coordinated fashion with players, policies, and programs that use a comprehensive, socio-ecological approach across sectors, as well as up and down over levels, with a flexible skeleton of structures to support it and an efficient set of communication linkages to maintain and change it: in other words, effective v/h integration of structures and process of multiple intervention programs. Examination of examples, both in health and other sectors raise important issues to consider in the areas of research, practice, and policy.

### Research/evaluation

Evaluation of multiple intervention programs is often a manifold process that requires integrating the program monitoring and evaluation design into the whole program planning process [12]. Childhood overweight/obesity interventions and intervention studies involving v/h integration are no exception. Standard multiple intervention program evaluation issues relevant to v/h integration include: drawing on the intervention's theory and evidence base to select and design an evaluation framework; developing a range of process and outcome indicators to capture change at various socio-ecological system levels targeted by multiple intervention programs; identifying the synergistic effects anticipated as a result of targeting more than one system level and developing indicators to capture them; developing tools that permit data collection across organizations, levels, and systems; developing data collection tools to capture coordinated and synergistic effects, not merely additive effects; and using ongoing monitoring mechanisms for feedback and adjustment [12].

Use of a conceptual framework such as the one above helps determine not only which interventions to consider and who to involve, but also what levels are employed, where synergies may lie, and with which stakeholders to coordinate measurement tools and activities. It may also serve as a tool to determine the extent to which integration is achieved in future initiatives, as well as a tool to enhance overall evaluation design. The spaces in between levels and sectors provide the routes for process-the networks, relationships, and interactions among the people involved in the different levels and sectors. To put such a framework into place, and use it to develop and maintain a well-functioning, collaborative network, requires both the development of relationships and allocation of time and resources to nurture and preserve them [23]. These too require evaluation, which needs to be planned at the start as it could get lost in the complexity of the components later on [18].

The framework above reminds us not only to consider development of indicators of outcome in terms of change in childhood overweight/obesity indicators, but in terms of process indicators of integration. Based on the examples and application to the framework, some possible evaluation indicators of successful, sound integration are presented in Table 1.

**Table 1:** Evaluation Indicators.

Program Element	Indicators
**Integration Structure**	Quantity-how much integration has occurred relative to the amount originally specified?

	Who is involved-are all the relevant sectors and jurisdictions represented?

	Level of support-are representatives merely seen to be at the table or are they truly involved (e.g., number of meetings attended, number of presentations made)?

	Financial commitment-is it sufficient to meet needs for interventions of sufficient intensity? Are there ties into funding allocation mechanisms; is funding offered to be sustained, renewed, or a one-time allocation?

	Political will-is there access and approval by senior policy makers?

	Bureaucratic will-is there access and approval by senior decision makers? Commitment of other resources-are time, people, and physical support in place?

	Sustainability-are the necessary conditions met?

	Integration criteria-have they been sufficiently met to merit further funding?

**Integration Process**	What is the quality of the integration: smooth, responsive to change and context, collaborative?

	How are stakeholders involved; what is their level of commitment, resources, investment? What are the mechanisms for approval, involvement?

	Does information flow both top down and bottom up?

	Does information flow in a timely manner?

	What are the facilitators and barriers to the process? Have they been addressed?

	Who is accountable for the intervention(s)? Is it shared over by the group, or is it held by individual sectors and levels? With either scenario, how are decisions made, and by whom? Do all stakeholders feel they have some ownership?

	How does the integrated program/intervention manage the boundaries - the process of managing a fully 'integrated' intervention process is highly complex and dynamic.

While the framework and the indicators are a beginning step, some questions still remain as to how best to evaluate interventions using v/h integration, as well as of the contribution of the v/h integration itself in a more dynamic fashion. We need to be able to capture synergies of intervention processes and outcomes among levels and sectors, as well as determine how well the integration and its underlying processes are being maintained over time. Much more systematic examination needs to be conducted to understand the shorter- and longer-term trade-offs between horizontally-directed and vertically-oriented approaches [17] and the resulting contributions of their synergies. Further, evaluation of v/h integrated strategies for childhood overweight/obesity requires more than measurement of the impact of the strategies in terms of overweight/obesity-related outcomes, although the ultimate outcomes of healthy children would still remain the focus. Evaluation also requires measurement of the success of the integration itself, to determine the success of the v/h activities as well as the degree of coordination among stakeholders. The framework and indicators bend to those purposes. Future research and evaluation, however, also need to determine whether the processes of integration are contributing toward the outcome, and if so, how much of the outcome can be attributed to them, both positively and negatively. For example, will the mere fact that stakeholders are willing to expend the resources and effort required to work together in a complex fashion lead to intensified efforts by program advocates, accounting for results beyond synergies and broad determinant coverage?

### Policy

The potential involvement of many stakeholders, from multiple jurisdictions and sectors, including private (*e.g*., food service industry in schools) and non-profit (community advocacy groups) as well as public sector, calls for examination and alignment of policy.

Structural issues of management and governance are critical to the development of such v/h integration. Administrative and governance structures, as well as funding mechanisms, are necessary to support such work. Some successful approaches have included working within existing governance structures, such as occurred with the Province of Ontario's Tobacco Control Strategy [37]. In the 1990's, funding was made available for tobacco initiatives. To access the provincial money, public health departments at the municipal level were asked to submit proposals on reducing/preventing tobacco use in their jurisdiction. Work proposed had to include involvement with other departments in their municipalities to develop an effective plan that supported horizontal integration. In British Columbia, as part of the government's 2005/06-2007/08 strategic plan, the premier asked every department to indicate how they can contribute to promoting the public's health [38]. A similar approach could be taken for the prevention of childhood overweight/obesity.

Further policy work to consider for childhood overweight/obesity intervention is the possibility of integration similar to what has been done with chronic disease initiatives. Initiatives aimed at different, yet related health issues benefit by joining forces (pooling resources, contributing to multiple health outcomes), thereby avoiding duplication and/or conflicting approaches among programs. For instance, provincial and local Active Living initiatives may collaborate with federal Environmental Health initiatives-for example, learning about nature, creating community gardens or trails. Such initiatives cross traditional boundaries with outcomes of increasing physical activity, healthy eating, and stress management, for example, while fostering appreciation of the broader environment. How can these initiatives best be sustained and fully integrated into prevention programs?

### Practice

The conduct of the interventions themselves requires skilled practitioners at many levels operating in coordination with each other. With childhood obesity, many types of practice expertise are involved. Practitioners from the fields of education, health, social welfare, psychology and counselling, nutrition, recreation, fitness, and urban planning could all be involved. To maintain this coordination, and keep activities true to sectoral and jurisdictional mandates requires effective relationships and timely communication and feedback through linkages and networks. What kind of networks need to be established, and of what quality, in order for v/h integration to function?

Members of such types of integrated networks could include a wide variety of partners (non-profit, non-governmental organizations, private sector, government, research groups, professional groups) forming broad coalitions (indeed, coalitions of coalitions) and funding consortia at all government levels (*e.g*., Canadian municipal, provincial/territorial, and federal levels). Networks will need to be sufficiently flexible and extensive to meet the needs of a variety of partners. While most such groups may be initiated at the national level, the tobacco experience suggests that local advocacy groups may also work to start integrated, system-wide change. Supports will need to be in place to facilitate both approaches, given the likely issues around ownership, interaction preferences, and mandated realities. Communication and network maintenance will be an important process function.

How the program manages the linkages among sectors and levels will have a significant impact on its success, and is a significant challenge. Beyond well-documented partnership and coalition-building relationship skills [14], another important component is accountability-who is accountable to whom within the various structures of the integrated program, and how do they relate to the program overall? Who is accountable for turning policy into action [21]? Is there anyone accountable over the whole project to ensure the integrity of the intervention and maintain collaboration and linkages, without other role conflicts [39]? Such accountability includes consideration of how the linkages are best developed, evaluated, and then strengthened or eliminated, based on the evaluation. In other words, are there other players who work in those spaces between framework cells, and can assist with and augment the collaboration and integration functions?

We know that overweight/obesity has traditionally been intransigent to short-term intervention, as previous work involving long-term follow-up has shown [15,24]; and further, that sustainability of obesity interventions themselves has been problematic [40,24]. Public health interventions aimed at childhood overweight/obesity may well require full v/h integration to meet its goals.

If h/v integration occurs, childhood overweight/obesity reduction outcomes may become more maintainable. Synergies produced as interventions from various levels and sectors working off of each other's success hopefully will result in increasing effectiveness of prevention and reduction efforts. The emphasis on integrated causal factors and on involving stakeholders, including nonprofit sector obesity advocacy groups, may result in reduced stigma from society and from health practice, particularly for children's programming [41]. In the long term, v/h integration can lead to new ways in how people relate to their environment, and in how their environment relates to them. The physical environment may become more activity friendly, technology may be reworked, media messages may change, and food service opportunities evolve. Linkages and networks begun now may provide ongoing benefits as technology advances and our environmental context changes. Such linkages will allow for modulation of approaches at both micro and macro levels, and foster the innovation required for sustainability.

### Some final thoughts

Vertically and horizontally integrated obesity intervention could play a role in helping understand the processes of such complex integrations. For example, from our perspective, one subset of processes may be subsumable under the construct of 'integrity.' Integrity conveys a notion of consistency and cohesiveness. While the term 'integrity' can be defined as 'moral uprightness, honesty' or 'wholeness, completeness' [42], it also means: 'soundness; unimpaired or uncorrupted condition' [42]. The latter definition reflects the sense with which we use it: the sense of the soundness or integrity of a true arrow. Systems integrity uses the concept of judging the integrity of systems in terms of their ability to achieve their goals via perceived and actual consistency of actions, values, methods, measures, principles, expectations, and outcomes. Here, coherence, stability, unity, and wholeness are the key components of integrity, including lack of impairment or degradation by disruptions in internal or external environments. However, more work needs to be done on delineating this construct, and how it may or may not differ from other aspects of integration. Obesity work could contribute to this understanding of what may be a crucial function.

More importantly perhaps, what v/h integration may achieve is a different way of thinking about the issue of childhood obesity as a society. We think about tobacco use much differently now than a decade ago, and our expectations around its use are also different. In relation to preventing overweight/obesity, we need to think differently about the environments we create, how we move around them, and what opportunities our children have to be socially nourished, physically active, and eat well, now and in the future.

## Summary

Both v/h integration across sectors and over system levels are needed to fully support multiple intervention programs of the complexity and scope required by obesity issues. V/h integration requires attention to system integrity and process. A conceptual framework is needed to support policy alignment, multi-level evaluation, and ongoing coordination of people at the front lines of practice. However, use of such tools and of achieving integration may enhance sustainability, increase effectiveness of prevention and reduction efforts, decrease stigmatization, and lead to new ways to relate the environment to people and people to the environment for better health for children.

## Competing interests

The authors declare that they have no competing interests.

## Authors' contributions

All authors contributed to the literature review, conceptual development and writing of the article. All authors read and approved the final manuscript.
